# Failure of Mandibular Distraction Osteogenesis in Klippel- Feil Syndrome- 4: A Case Report of a Rare Syndromic Robin Sequence

**DOI:** 10.1177/10556656231220852

**Published:** 2023-12-13

**Authors:** Emma Yanko, Brandon Spink, Craig Gendron

**Affiliations:** 1 College of Medicine, 12371 University of Saskatchewan, Canada; 2 Department of Surgery, Division of Plastic Surgery, College of Medicine, 12371 University of Saskatchewan, Canada

**Keywords:** craniofacial morphology, mandible, outcomes, Pierre Robin sequence, upper airway obstruction

## Abstract

Klippel-Feil syndrome-4 (KFS4), a rare autosomal recessive form of Klippel- Feil syndrome, is characterized by facial dysmorphism, nemaline myopathy, and short stature. Only 10 cases of KFS4 have been previously published in the literature. We report a novel case of a 1- month-old girl with known KFS4 and Robin Sequence (RS). At 2 months old, she underwent bilateral mandibular distraction osteogenesis to correct significant airway obstruction. Despite adequate mandibular advancement, the patient failed extubation twice and eventually required a tracheostomy. Due to the multiple anomalies present in KFS4, mandibular distraction osteogenesis may have a decreased likelihood of surgical success.

## Introduction

Robin sequence (RS), previously known as Pierre Robin Sequence, is a rare congenital condition first described in 1923 by French dentist, Pierre Robin.^1^ It is characterized by micrognathia, glossoptosis, and upper airway obstruction. Cleft palate, while not considered a mandatory characteristic of the condition, is present in up to 90% of patients with RS.^2^ RS can occur in isolation, however, 50% of patients have an associated syndromic condition or chromosomal abnormality.^3^ To date, more than 50 different syndromes have been associated with RS.^4^

Klippel-Feil syndrome (KFS), first reported in 1912 by Maurice Klippel and Andre Feil, is a rare multisystem disease characterized by the congenital fusion of two or more vertebrae in the cervical spine.^5^ These patients typically present with a triad of limited cervical range of motion, an abnormally short neck, and a low posterior hairline.^6^ Other spinal and extraspinal anomalies have been reported in patients with KFS, including Sprengel deformity, congenital scoliosis, and several visceral pathologies.^6,7^ Klippel-Feil syndrome-4 (KFS4; OMIM#616549) is an autosomal recessive form of KFS caused by biallelic pathogenic variants in MYO18B.^8,9^ KFS4 is a rare condition; to our knowledge, only 10 cases have been reported in the literature.^8,9^ Unlike KFS, these patients often present with nemaline myopathy, facial dysmorphism, and short stature.^8^ Although micrognathia has been reported as a craniofacial phenotype in patients with KFS4, mandibular distraction osteogenesis for concurrent RS has not been described to our knowledge.^10^

We report a novel case of a 1-month-old girl with KFS4 and RS who underwent bilateral mandibular distraction osteogenesis (MDO) with internal Micro Zurich distraction devices (KLS Martin) for correction of RS.

## Case Report

A 1-month-old girl with previously diagnosed KFS4 and RS was admitted to the hospital with upper airway obstruction and failure to thrive.

Previously, at two weeks of age, the patient underwent whole exome sequencing due to a constellation of anomalies, including facial dysmorphism with severe micrognathia, retrognathia, and glossoptosis. Other findings included nemaline myopathy causing hypotonia of the upper extremities, and severe scoliosis (Table 1). The genetic testing results showed that the patient is a compound heterozygote for two variants in the MYO18B gene, leading to the diagnosis of Klippel-Feil syndrome-4 (KFS4).

**Table 1.** Known Anomalies in Case Report Patient Diagnosed with Klippel-Feil Syndrome-4 and Robin Sequence.

System or Anatomic Area	Findings
Craniofacial	Glossoptosis Micrognathia Retrognathia U-shaped cleft palate Microcephaly
Musculoskeletal	Scoliosis at the level of thoracolumbar vertebrae Thoracic spine fusion Bilateral hip subluxation Club feet Nemaline myopathy, with hypotonia of the upper extremities Arthrogryposis of the hands
Respiratory	Tracheomalacia Mild restrictive lung disease secondary to scoliosis
Cardiovascular	Small atrial septal defect Potential aberrant subclavian artery

Regarding her upper airway obstruction, the patient was evaluated by a multidisciplinary team. This included pediatric plastic surgery, otolaryngology, anesthesia, and respirology. A full night unobserved smart monitor study on 0.2 L/ minute of low-flow oxygen was completed, and the patient was classified with moderate obstructive apneas. Nasal laryngoscopy demonstrated glossoptosis and a normal-looking larynx and vocal cords. Flexible scope demonstrated mild, distal tracheomalacia, no concerning tracheal stenosis, and some pulsatility along the right mainstem bronchus, potentially due to an aberrant subclavian artery in the area.

**Figure 1.**
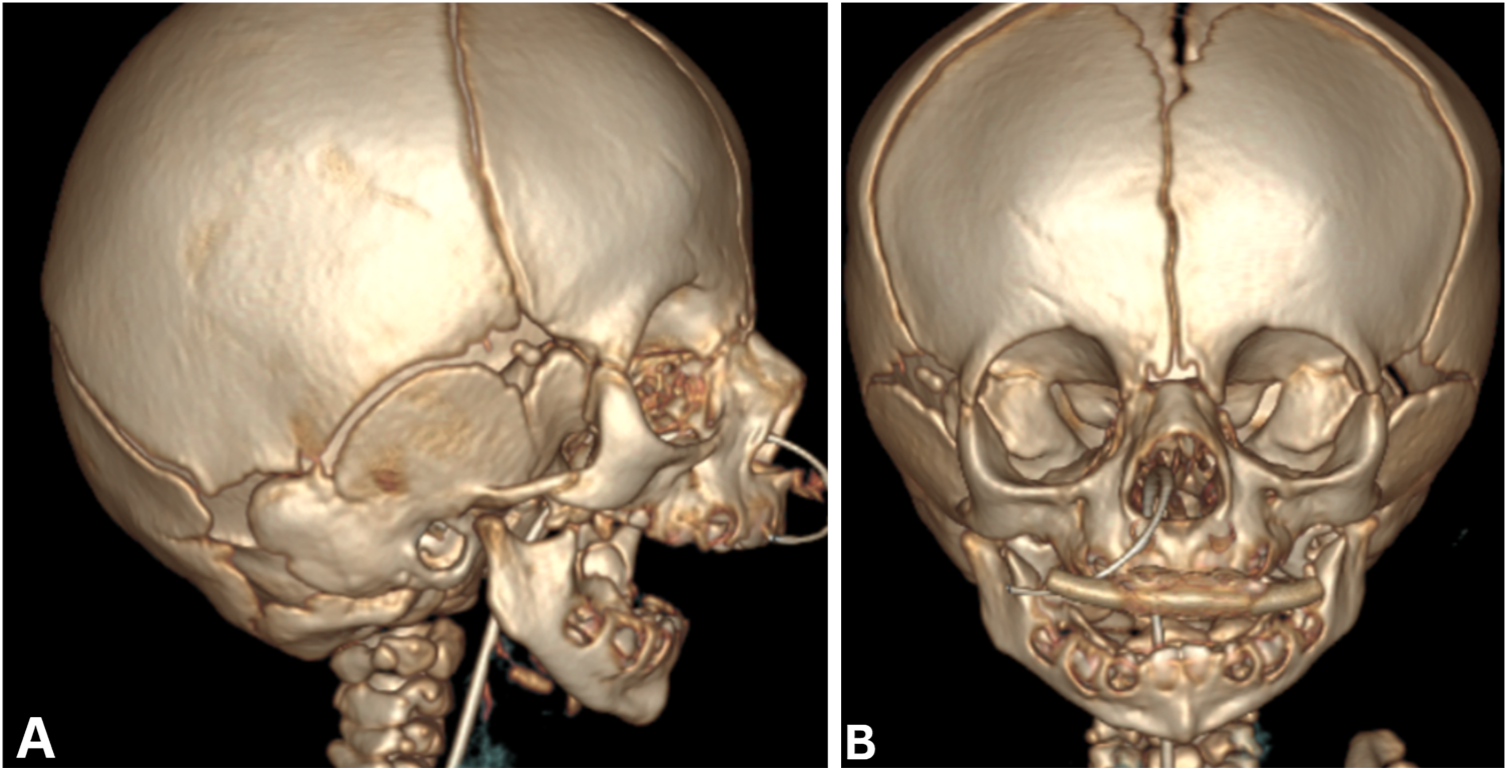
Computed tomography image reconstruction: lateral (A), anterior (B) demonstrating severe micro-/retrognathia.

**Figure 2.**
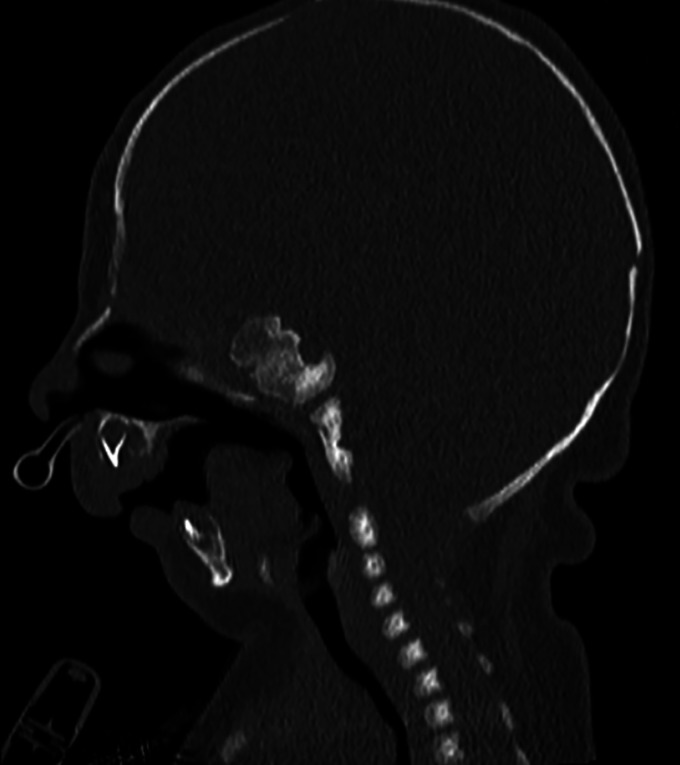
Computed tomography: sagittal view demonstrating severe micro-/retrognathia.

A computed tomography (CT) head showed severe micrognathia and retrognathia, with a large anterior-posterior (AP) discrepancy between the maxillary and mandibular dentition, as well as glossoptosis (Figures 1 and 2). These findings were concluded to be the primary cause of the patient's severe upper airway obstruction; given this, the patient was determined to be a candidate for mandibular distraction osteogenesis.

At baseline, the patient had upper airway obstruction on clinical examination and required high-flow nasal cannulation. Nasogastric (NG) tube feeding, which the patient had been on since postnatal discharge from the neonatal intensive care unit (NICU), was continued to prevent further aspiration and promote weight gain. Antibiotics were initiated for suspected aspiration pneumonitis.

**Figure 3.**
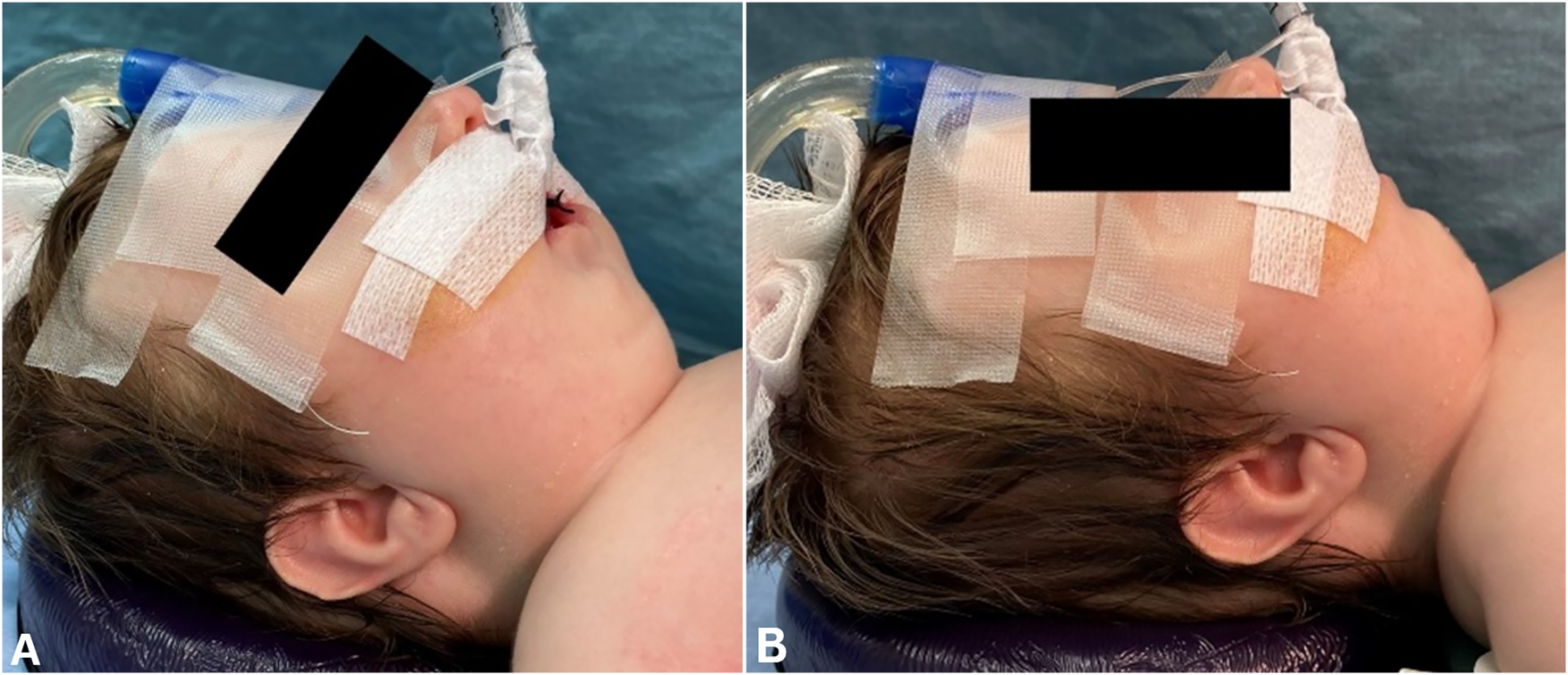
Pre- mandibular distraction osteogenesis photographs (A and B) demonstrating severe micro-/retrognathia.

**Figure 4.**
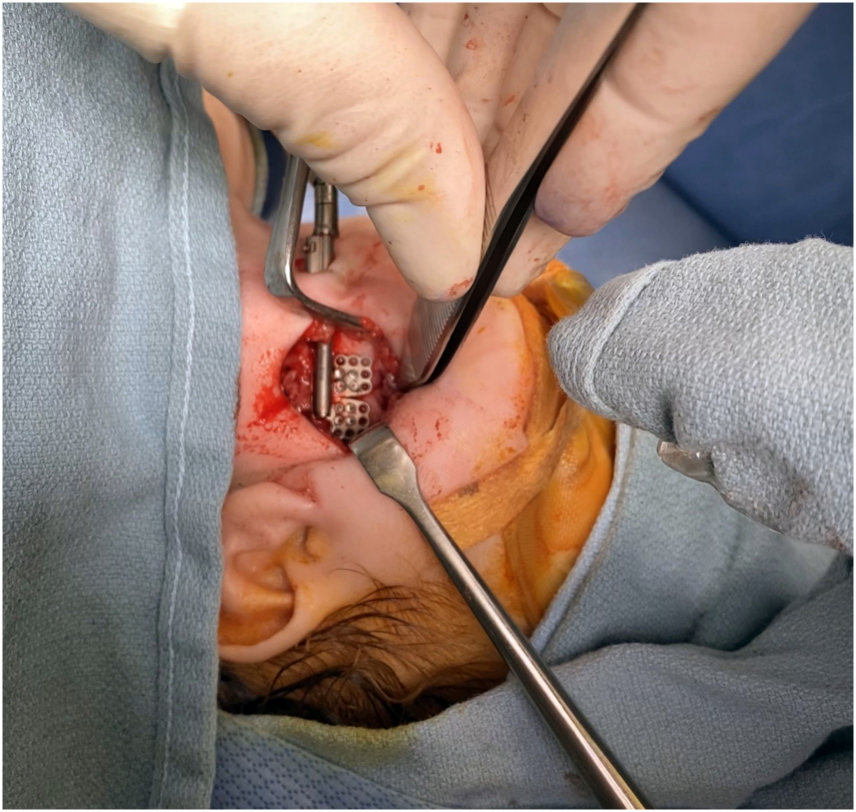
Placement of internal Micro Zurich distraction devices (KLS Martin).

After a discussion of the risk and benefits of MDO, the patient's parents opted to proceed with surgery in hopes of avoiding tracheostomy despite the increased chance of surgical failure. At two months and four days of age, the patient underwent bilateral mandibular distraction with the placement of internal Micro Zurich distraction devices (KLS Martin) as well as gastrostomy tube placement (Figures 3 and 4). Regarding the surgical techniques used, bilateral Risdon incisions were made 1.5 cm below the mandibular border. Subperiosteal dissection of the mandible was carried out, revealing the angle of the mandible as well as the area to place the distractor. The Sonopet(®) ultrasonic bone aspirator (Stryker(®), Kalamazoo, MI) was then used to cut the bone and make a corticotomy in the lateral and inferior surface of the bone. After securing the distractors bilaterally, the bone was fractured by distracting the devices approximately 2-3 mm, using a 2 mm osteotome, and completing posterior corticotomy. The distractors were then backed off to the starting position.

Post-operatively, the patient remained intubated and was sent to the NICU for post-operative healing and mandibular advancement. Starting on postoperative day one, the distractors were turned daily for 15.5 days (distraction rate of 1.2 mm/day). Leading to a total of 18.6 mm of distraction. Before hardware removal, twenty weeks were allowed for consolidation. After completion of MDO, there appeared to be mild retrognathia and the glossoptosis was corrected.

**Figure 5.**
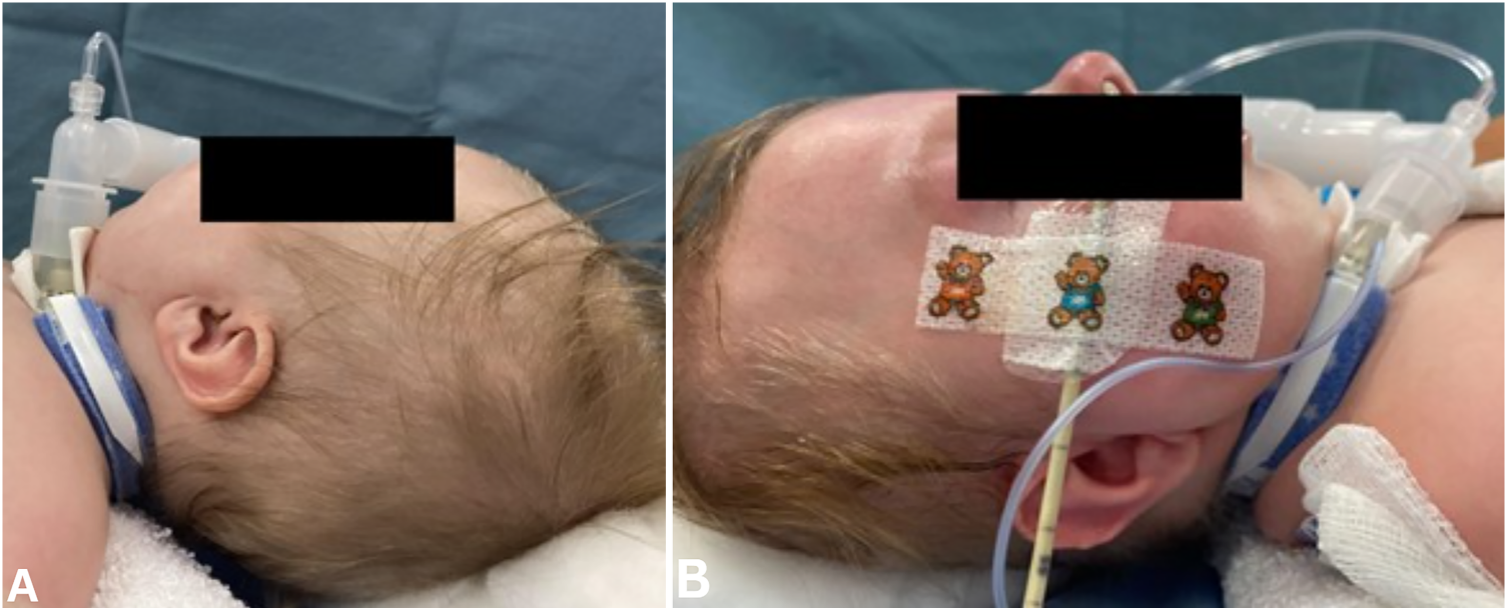
Post mandibular distraction osteogenesis hardware removal photograph demonstrating clinically improved micro-/retrognathia (A and B).

However, despite adequate mandibular advancement, the patient failed two extubation attempts due to ongoing upper airway obstruction. To minimize the complication risk associated with prolonged intubation, a tracheostomy was performed to secure the airway at four weeks post-MDO surgery. Mandibular distraction hardware removal occurred when the patient was six months old, and twenty weeks post MDO surgery (Figure 5).

At the time of writing, the patient is now 30 months old and continues to have a tracheostomy tube. She requires continued tracheostomy management for respiratory secretions.

## Discussion

Robin sequence is a craniofacial anomaly characterized by a collection of findings; these include micrognathia, glossoptosis, and, commonly cleft palate, which results in upper airway obstruction and feeding difficulties.^3,4,11^ RS may occur in isolation (iRS) or as a feature of other congenital syndromes, termed syndromic Robin Sequence (sRS). Patients with additional anomalies and/or chromosomal defects without an identified syndrome are classified into the Robin Sequence plus group (RS-plus). The clinical expression of RS is heterogeneous with the severity of clinical obstruction presenting on a spectrum.^11–13^ A patient with RS may be otherwise asymptomatic or demonstrate some degree of upper airway obstruction ranging from mild respiratory distress to overt obstruction requiring immediate intervention.

The management of RS is contingent on the severity of upper airway obstruction as well as other presenting comorbidities.^11,12^ There have been multiple algorithms developed to aid in treatment decision-making for patients with RS, though no formal consensus has been reached.^11,13–15^ The majority of patients with RS see spontaneous improvement or resolution of syndrome sequelae within the first two years of life as both the mandible and upper airway increase in size.^16,17^ Concurrent feeding difficulties and aspiration risk can often be managed effectively with upright feeding techniques, modification of the nipple for bottle feeding, or temporary tube feeding.^11^ Conservative airway management is also reasonable in these patients and includes close observation and prone positioning. In addition, the use of an orthodontic airway plate, such as the pre-epiglottic baton plate (PEBP), is a valuable treatment method that may be used in an attempt to avoid more invasive treatment.^18,19^ Evidence suggests that an orthodontic airway plate may offer similar airway-related and feeding-related improvements in patients with RS when compared to MDO.^18^

When conservative management is unsuccessful or the patient is severely symptomatic, which often is the case with syndromic RS, surgical intervention is indicated. Options for surgical management include tongue-lip adhesion, mandibular distraction osteogenesis, and tracheostomy.^11,12,20^ Mandibular distraction osteogenesis in the treatment of symptomatic micrognathia was first described in 1992 by McCarthy et al.^21^ MDO has become a mainstay in the initial treatment of patients with RS associated with severe airway obstruction, obstructive sleep apnea, or oral feeding difficulties and has a success rate of avoiding tracheostomy ranging from 84- 100%.^11,13,22–27^ Mandibular distraction osteogenesis is also considered an effective method to facilitate decannulation in patients with RS who underwent tracheostomy initially.^3,11^ The procedure works by lengthening the mandible in a forward direction, consequently pulling the tongue anteriorly through its muscular attachments on the lingual surface of the mandible to increase the retrolingual airway.^11^ Multiple techniques for MDO have been described, though oblique osteotomies at the angle of the mandible remain the most common approach and were used in this case.^20^ Compared to alternative surgical options, MDO is a relatively safe procedure with lower rates of long-term complications.^3,11^ In contrast, tracheostomy offers a definitive treatment for upper airway obstruction but has associated risks of accidental decannulation or mucous plugging.^3^ There is also potential long-term morbidity related to peristomal scarring and tracheal erosion in addition to the need for long-term maintenance and home care.^3^

The patient presented in this case has Klippel-Feil syndrome-4, an exceedingly rare disorder associated with multiple anomalies (Table 1). Although KFS4 is associated with facial dysmorphism, micrognathia has only been described in 3 of the 10 known KFS4 cases reported in the literature.^8,9^ Due to the severity of this patient's airway obstruction symptoms, surgical management was necessary in this case. Unfortunately, although an adequate mandibular advancement of 18.6 mm was achieved, the patient failed extubation twice due to ongoing airway obstruction, resulting in the need for a tracheostomy. As cited earlier, MDO is often successful in correcting upper airway obstruction in RS sequence. However, many of these studies only included a small percentage of patients with sRS. It has been suggested that patients with sRS have poorer outcomes from MDO than those with iRS.^11^ The comorbidities leading to the decreased surgical success of MDO in RS treatment have not been well described. It has been reported that in patients with multiple levels of airway obstruction, such as laryngomalacia, and tracheomalacia, MDO may not successfully relieve the obstruction.^14^ In this patient's case, ongoing airway obstruction after MDO may have been due to one or more of the following factors: severe scoliosis, mild tracheomalacia, and nemaline myopathy. Given the numerous anomalies associated with syndromic RS and RS-plus, we would be remiss if we did not mention the necessity of early involvement of a multidisciplinary team in the assessment and management of this condition.^28^ As one example, specialists in pediatric respirology and neurology are important in evaluating cardiopulmonary and neurocognitive function to determine whether airway compromise is the result of multilevel obstruction or central apnea.^29^

The decision to proceed with MDO in this patient, despite an increased chance of failure, was due to multiple factors. Firstly, the patient had a classic presentation of RS and upper airway obstruction secondary to this. Although this patient did have mild tracheomalacia and mild restrictive lung disease secondary to severe scoliosis, these factors were not thought to be the main cause of the patient's airway obstruction. In addition, the patient's parents were aware that given her comorbidities, there would be an increased likelihood that she would require a tracheostomy after MDO. However, the patient's parents opted to proceed with the initial MDO despite the increased risk of surgical failure. Although the patient ultimately required a tracheostomy, the patient's parents are pleased with the improved anatomic appearance, report increased ease of tracheotomy care given the improved micro/retrognathia, and are relieved that the medical team made every effort for their child before resorting to tracheostomy.

## Conclusion

Mandibular distraction osteogenesis is often successful in treating patients with Robin Sequence who have failed conservative therapy. However, co-morbidities in syndromic Robin sequence may affect the surgical success of MDO. In the context of Klippel-Feil syndrome-4, these co-morbidities include concurrent airway, respiratory and musculoskeletal anomalies. Presently, there is a lack of comprehensive understanding regarding the impact of co-morbidities on the effectiveness of mandibular distraction osteogenesis. Further research is needed to clarify this matter. It is essential to consider specific patient and family factors coupled with a multidisciplinary assessment of the patient to evaluate the potential risks and advantages associated with mandibular distraction osteogenesis in these individuals.
